# Subverting Host Cell P21-Activated Kinase: A Case of Convergent Evolution across Pathogens

**DOI:** 10.3390/pathogens6020017

**Published:** 2017-04-21

**Authors:** Simona John von Freyend, Terry Kwok-Schuelein, Hans J. Netter, Gholamreza Haqshenas, Jean-Philippe Semblat, Christian Doerig

**Affiliations:** 1 Infection and Immunity Program, Monash Biomedicine Discovery Institute and Department of Microbiology, Monash University, Melbourne, Victoria 3800, Australia; terry.kwok@monash.edu (T.K.-S.); Hans.Netter@mh.org.au (H.J.N.); gholamreza.haqshenas@monash.edu (G.H.); 2 Cancer Program, Monash Biomedicine Discovery Institute, Department of Biochemistry and Molecular Biology, Monash University, Melbourne, Victoria 3800, Australia; 3 Victorian Infectious Diseases Reference Laboratory, Melbourne Health, The Peter Doherty Institute, Melbourne, Victoria 3000, Australia; 4 Inserm UMR-S 1134, Université Paris Diderot, Paris 75013, France; jean-philippe.semblat@inserm.fr

**Keywords:** signalling, virus, bacteria, parasite, kinase, host-pathogen interactions

## Abstract

Intracellular pathogens have evolved a wide range of strategies to not only escape from the immune systems of their hosts, but also to directly exploit a variety of host factors to facilitate the infection process. One such strategy is to subvert host cell signalling pathways to the advantage of the pathogen. Recent research has highlighted that the human serine/threonine kinase PAK, or p21-activated kinase, is a central component of host-pathogen interactions in many infection systems involving viruses, bacteria, and eukaryotic pathogens. PAK paralogues are found in most mammalian tissues, where they play vital roles in a wide range of functions. The role of PAKs in cell proliferation and survival, and their involvement in a number of cancers, is of great interest in the context of drug discovery. In this review we discuss the latest insights into the surprisingly central role human PAK1 plays for the infection by such different infectious disease agents as viruses, bacteria, and parasitic protists. It is our intention to open serious discussion on the applicability of PAK inhibitors for the treatment, not only of neoplastic diseases, which is currently the primary objective of drug discovery research targeting these enzymes, but also of a wide range of infectious diseases.

## 1. Introduction

The serine/threonine kinase p21-activated kinase (PAK) is found in most eukaryotic systems and plays a central role in diverse cellular processes. Several paralogues of PAK have been discovered, and are classified based on sequence and domain organisation into Group I PAKs, which include PAK1 (see BOX 1), PAK2, and PAK3, together with Group II PAKs, which include PAK4, PAK5, and PAK6. Group I PAKs are evolutionarily highly conserved and have a wide range of cellular functions [1], including cell survival, cytoskeletal organization, cell cycle progression, as well as important scaffold functions [2,3,4]. PAKs have overlapping as well as distinct functions. We focus this review on PAK1—and to a lesser extent on PAK2—for which the largest body of data is available.

BOX 1 **Domain organization and activation mechanism of PAK1.**

**Figure 1:**
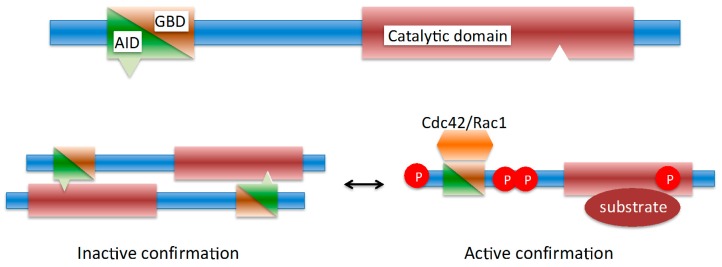
Schematic of the activation mechanism of PAK1.

PAKs generally contain a carboxyterminal kinase domain, with a single activating phosphorylation site. In human PAK1, this activating phosphorylation site is at serine residue 141. The N-terminal part of PAKs comprises an auto-inhibitory domain (AID), which partially overlaps with the p21-GTPase-binding domain (GBD) (Figure 1). In its inactive state PAK1 exists as a homodimer, with the auto-inhibitory domain bound to the kinase domain of another PAK1 molecule. Activation of PAK1 occurs through binding to the active small GTPases Rac1 and Cdc42. Binding of Rac1 and Cdc42 to the N-terminal p21-GTPase-binding domain leads to a reorganization of the homodimer and subsequent autophosphorylation of PAK1 on serine 141. The binding of a substrate to the kinase domain of phosphorylated PAK1 results in dissociation of PAK1 dimers into monomers. Other molecules, like membrane lipids and receptor agonists, can also activate PAK 1 either alternatively or in conjunction with Rac1 and Cdc42 [2,5,6]. For a more detailed description of PAK activation mechanisms refer to [3,4].

Most PAK paralogues, but in particular PAK1 and PAK4, show increased activity in several human cancers, and many tumour types become dependent on PAK signalling and are thus hypersensitive to PAK inhibition [4]. Even though PAKs have a central role in signalling, PAK1 knock-out mice are viable and show only relatively mild mast cell and macrophage defects, as well as impaired glucose homeostasis [4,7,8]. This lends hopes that PAK-directed treatments will be a useful therapeutic approach against cancer in the future [9].

Human PAK1 also plays an important role for a wide range of disease-causing pathogens. To investigate whether human PAK1 might also act as a useful therapeutic target in infectious diseases it will be highly beneficial to understand the role PAK1 plays in the successful completion of the life cycles of the causative organisms.

Pathogens have to overcome intrinsic defence mechanisms of their host cells, as well as to evade the host innate and adaptive immune responses. Both PAK1 and 2 have roles in various host-driven responses to pathogen infection, including anti-pathogen signalling [10] and immune regulation [11,12].

To overcome host defences, many pathogens encode kinases and other virulence factors to induce changes in the host cell to promote their own life cycle, replication and/or spread. In addition, it is now well established that pathogens can take advantage of host cell pathways to modulate the activity of their own proteins and/or host proteins. Pathogens can moreover directly interact with host kinases and hijack them for their own advantage [13,14,15]. Due to the ability of PAKs to regulate crucial cellular processes such as cytoskeleton dynamics, adhesion, motility, or apoptosis [16,17], pathogens of many taxons have developed ways to target functions of PAK in their host cells.

**Figure 2:**
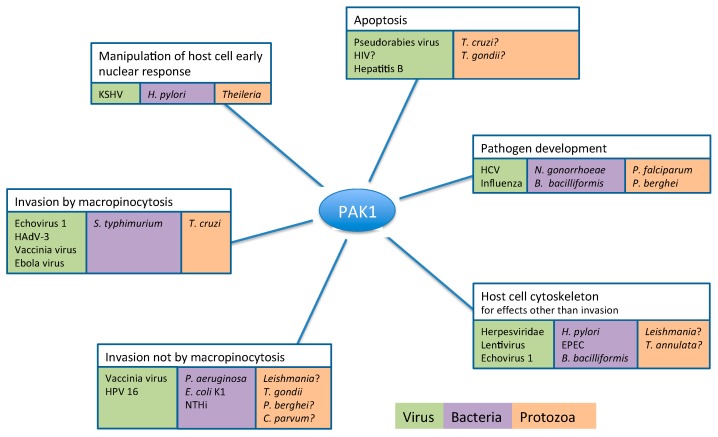
Pathogens of all three classes—virus, bacteria and parasitic protists—modulate host PAK1 signalling for a diverse range of purposes. For pathogens listed with a “?” there is so far no direct evidence available for the involvement of PAK1 in diverting host cell signalling to the pathogen’s advantage. However, circumstantial evidence points to a likely involvement of host PAK1 in the respective processes, which led to inclusion in this review.

Here we describe the important roles that host PAK1 (and PAK2, see BOX 2) plays during infection by viruses, bacteria, and parasitic protists, examining how pathogens target PAK1 signalling to interact with their host cell at several stages of their infectious cycle to promote entry into, survival and replication within, and egress from their host cell (see Figure 2 and Table 1 for a summary). Furthermore, not all pathogens that influence host signalling events reside within their host cells. This review therefore also includes several examples of extracellular bacterial pathogens that affect PAK1 signalling in human cells during the course of infection. See BOX 3 for a brief description of all pathogens included in this review and the diseases they cause. Unless otherwise stated the studies cited investigate PAK1 in the human host.

BOX 2 **The role of host PAK2 in pathogen infection.**

Research on p21-activated kinases has traditionally been focused mainly on PAK1. However, some evidence exists that PAK2 likewise plays an important role in infectious disease establishment in the host tissue. In this Box we summarise the available data in regard to PAK2’s role in infectious disease development and progression.

PAK2 shows a 93% sequence homology to PAK1 in its catalytic domain, with a 72% overall sequence identity. We would like to draw attention to a particular experimental issue: Available antibodies, especially when directed against specific phosphorylation sites in PAKs, often cannot distinguish between PAK I paralogues. It is therefore not always possible to determine whether one observes PAK1, PAK2, or PAK3 phosphorylation in an antibody-based experiment. This needs to be taken into account when researching functions and involvement of specific PAKs, as well as when reviewing data. Indeed, some pathogens have been found to target not only PAK1 but also PAK2, examples of which are discussed below.

**Herpesviridae.** The viral US3 kinase activates both PAK1 and PAK2, which leads to rearrangements of the cytoskeleton in order to enhance intracellular virus spread [18]. Disassembly of stress fibres is PAK2-mediated, in contrast to the formation of cell projections which is controlled by PAK1 [19], indicating that different group I PAK paralogues can have distinct biological activities. **Lentiviruses** (human immunodeficiency virus type 1, simian immunodeficiency virus) express the auxiliary protein Nef (Negative factor) during the early stages of the viral life cycle. During the viral life cycle, Nef functions as a molecular adaptor [20] and interacts with host kinases of the Src family [21]. Nef is a critical factor for efficient virus replication in the infected host and for disease progression. Both PAK1 and PAK2 were found to be associated with Nef [22], yet in a siRNA approach only PAK1 but not PAK2 knock down inhibited HIV infection [23]. PAK2 however, is recruited by myristylated Nef into lipid rafts in the context of a multiprotein complex, possibly promoting the interaction with GTP-bound GTPases [24,25] involved in PAK2 activation [26]. PAK2 recruitment by Nef leads to an increased phosphorylation of a key signalling molecule, merlin [27], and the deregulation of cofilin and rearrangement of the cytoskeleton, with severe effects on cell motility and chemotaxis [28]. The host cell actin dynamics are further inhibited by the interaction of PAK2 with Nef and the excocyst protein complex (EXOC), which is involved in vesicular transport and actin remodelling [29]. Just like PAK1, PAK2 activation by Nef likewise requires phosphatidylinositol 3-kinase (PI3-kinase) and leads to the inactivation of the pro-apoptotic protein Bad [30].

The **enteropathogenic *E. coli* (EPEC)** type III secretion system secretes multiple effector proteins into host intestinal epithelial cells. One of those, EspG, interacts with the Rac/Cdc42 (p21) binding domain of PAK1 [31,32] and PAK2 [32]. In in vitro kinase assays, purified EspG significantly increases the kinase activity of PAK2 (7.6 ± 2.5 folds) [32]. EspG has also been found to co-localize with PAK2 and the ADP-ribosylation factor (ARF) GTPase, ARF1, at the Golgi when ectopically expressed in HEK293A cells, raising the possibility that EspG might function as a molecular scaffold that links ARF GTPase activity with PAK-mediated signalling pathways at the Golgi [32]. In response to ***N. gonorrhoeae*** infection of HeLa cells, PAK2 activity increases several-fold [33]. This activation is rapid but transient: it occurs as early as 15 min post-infection but is significantly diminished by 90 min post-infection [33]. The transcription factor AP-1 is notably inhibited when the dominant-negative kinase-dead mutant of PAK2 is expressed ectopically *N. gonorrhoea*-infected cells. Ectopic expression of wild-type PAK2 on the other hand enhances the level of AP-1 activation induced by *N. gonorrhoeae* [33]. Hence, AP-1 activation in this system seems to depend on PAK1 activity (see text), as well as PAK2 activity.

## 2. Pathogens Subvert Host PAK1 for a Range of Functions

### 2.1. Role of PAK1 in Pathogen Entry into the Host Cell

#### 2.1.1. Pathogen Entry by Macropinocytosis

Macropinocytosis is a transiently activated pathway to capture extracellular fluid, nutrients, solutes, and cell debris [63]. This endocytic process is initiated by actin-driven membrane ruffling, which leads to the formation of lamellopodia, filopodia, blebs or circular ruffles. Macropinocytosis is usually activated by external ligands such as growth factors and phosphatidylserine-containing cell residues. PAK1 regulates macropinocytosis by changing the dynamics of the cytoskeleton, and is required for macropinosome closure through activation of the cellular factor CtBP-1/BARS [64]. Viruses, bacteria, and protozoa utilize macropinocytosis as an entry pathway into their host cells [65,66,67] (Table 2).

The amphotropic murine leukemia virus (A-MLV, family *Retroviridae*), uses macropinocytosis as the predominant entry pathway into host cells requiring Na^+^/H^+^ exchange activity, actin dynamics, Rac1, and PAK 1. The use of dominant negative Rac1 and PAK1 mutants reduced macropinocytosis and infection, in contrast to the outcomes achieved with a dominant positive Rac1 mutant, which stimulated the A-MLV infection of mouse embryo fibroblast cells [68]. Echovirus 1 (EV1), of the genus *Enterovirus* in the family *Picornaviridae* binds to the host cell via α2β1 integrin, followed by integrin clustering, then entry, which is clathrin- and caveolin-independent. EV1 infection is prevented by inhibitors of macropinocytosis, and the use of dominant-negative or highly kinase active PAK1 constructs demonstrated the PAK1 dependence of EV1 entry via the α2β1 integrin receptors. Knock-down experiments using specific siRNAs indictated that Rho GTPase Rac1 regulates EV1 infection and is possibly the upstream regulator of PAK1 [68]. The process follows the epidermal growth-factor (EGF)-dependent macropinocytic pathway with macropinosome closure depending on PAK1-dependent phosphorylation of the C-terminal binding protein 1 (CtBP1/BARS) [64]. Finally, the internalized vesicles enter a sorting pathway to caveosomes [69]. Another member of the family *Picornaviridae*, the Foot-and-Mouth Disease virus (FMDV), uses macropinocytosis as an alternative endocytic pathway in addition to the clathrin-mediated entry pathway. As shown in hamster and porcine kidney cells, FMDV entry via macropinocytosis does not require PI3K, but PAK1 activation is essential to allow the viral entry process to occur [70]. Less specifically defined interactions might allow Ebola virus entry into target cells. The virus (family *Filoviridae*) interacts with cellular C-type lectin receptors through glycans on the viral envelope glycoproteins. Alternatively, binding of phosphatidylserine in the viral envelope to cellular phosphatidylserine receptors supports viral entry, possibly mimicking the clearance of apoptotic debris for the activation of macropinocytosis. Ebola virus glycoprotein binding to cellular receptors seems to activate multiple macropinocytosis inducers in African Green monkey cells, including Rac1, Cdc42 and PAK1 [71]. PAK1 and CtBP/BARS have been identified as essential components in this process but the full pathway that triggers the Ebola virus-induced macropinocytosis remains to be identified [72,73]. Influenza A virus (family *Orthomyxoviridae*) uses the major envelope protein, hemagglutinin, for binding to sialic acids at the host cell surface followed by clathrin-mediated endocytosis. Similar to FMDV, influenza a virus uses macropinocytosis as an alternative entry pathway. PAK1 activity is required; the activation of PAK1 seems in this case to be independent of the typical upstream signal transducers Cdc42 and Rac1, but may require the tyrosine kinase Src [74].

Similar to the RNA viruses mentioned above, PAK1 is also an important factor for entry of DNA viruses by macropinocytosis. The Singapore Grouper Iridovirus (SGIV), family *Iridoviridae*, uses macropinocytosis as an alternative entry pathway in addition to clathrin-mediated endocytosis. Inhibition of PAK1 and Rac1 activity strongly inhibited SGIV entry in a grouper spleen cell line [75]. The uptake of the African Swine Fever virus (ASFV) (family *Asfarviridae*), a highly pathogenic DNA virus, is independent of the clathrin-mediated endocytic pathway, and primarily mediated by macropinocytosis as demonstrated with the African Green monkey and porcine cell lines. This includes the involvement of key features such as the induction of cellular membrane ruffles and blebs, actin rearrangements and the involvement of the Rac1-PAK1 pathway [76]. Among the *Adenoviridae*, human adenovirus B type 3 (HAdV-3) entry (reviewed in [77]) is mediated by the receptors CD46 and α_v_ integrins, and depends on macropinocytosis triggered by the virus-dependent activation of PAK1, and subsequent phosphorylation of the PAK1 effector CtBP-1 [78]. CtBP-1 is localized on the periphery of HAdV-3 containing macropinosomes, most likely playing a role in their fission and stabilization. In line with this observation, the inhibition of PAK1 activity prevented HAdV-3 infections by blocking endocytic uptake via macropinocytosis [79]. Vaccinia virus (family *Poxviridae*) exposes phosphatidylserine on its surface, thus mimicking the use of phosphatidylserine for the uptake of apoptotic bodies [67]. Macrophages recognize phosphatidylserine and internalize the virus by macropinocytosis, dependent on actin rearrangements and PAK1 activity [67]. The newly identified keratinocyte receptor MARCO for vaccinia virus may play a role in this process [80]. Differences were observed depending on the Vaccinia virus strain, with either the formation of filopodia induced by virus-dependent activation of host Cdc42 or by blebbing following host Rac1 activation [81]. Vaccina virus and a second poxvirus, Myxomavirus, exploit PAK1 at different stages during viral entry; PAK1 is required for Vaccinia entry, but for Myxomavirus at a post-entry stage [82].

Host cell entry via PAK-mediated macropinocytosis has also been observed with the bacterial pathogen *Salmonella* [83]. The type III secretion system (T3SS) of *Salmonella* Typhimurium (see BOX 3) induces macropinocytosis as a result of stimulation of dramatic membrane ruffling and actin rearrangement in host cells at the sites of bacterial attachment. SopE, one of the T3SS effector proteins [84], possesses properties of a mammalian guanine nucleotide exchange factor (GEF) of small Rho GTPases and is able to trigger membrane ruffling and activation of the host c-Jun NH_2_-terminal kinase (JNK) [85]. JNK activation by SopE takes place in a PAK-dependent manner, whereas expression of the kinase-defective mutant of PAK abrogates pathway activation. Moreover, SopE co-localises with PAK at membrane ruffles. It is plausible that SopE may be able to recruit PAK to membrane ruffles [86].

The trypomastigote stages of the parasitic protist *Trypanosoma cruzi* invade a large variety of nucleated cells, using multiple mechanisms such as host cell endocytosis/phagocytosis and active parasite invasion [87]. *T. cruzi* can also enter mammalian host cells by macropinocytosis [88]. The PAK1 inhibitor IPA-3, which inhibits group I PAKs (PAK1–3), but not PAK4–6 [89], impaired internalization of Y-strain trypomastigotes into peritoneal macrophages by 70%, and amastigote entry into peritoneal macrophages was reduced by 50% after PAK inhibition. Immunofluorescence experiments localized host cell PAK1 in the parasitophorous vacuole of infected peritoneal macrophages, while opsonized trypomastigote controls showed no colocalization with host PAK1 [88]. These data indicate that the activity of host cell PAK (or at least of an IPA-3 inhibitable activity) is essential for *T. cruzi* invasion by macropinocytic mechanisms. In addition, independent experiments showed that *T. cruzi* (Y strain) relies on actin filament formation during the invasion of host cells, as internalization of parasites was severely reduced when actin polymerization was inhibited by cytochalasin D [90,91,92]. The role of PAK1 in the control of actin cytoskeleton organization in mammalian cells is well established [93,94], and is consistent with the observation that the small GTPase Rac1, a well-established PAK1 activator, plays a role in host cell invasion by *T. cruzi* Y strain [95,96]. Despite this evidence, macropinocytosis does not seem to be the only or even the most common entry pathway for *T. cruzi* [97,98].

BOX 3 **The pathogenic organisms reviewed in this paper, in alphabetical order.**

***1.* *Viruses***

***Adenoviridae*** are non-enveloped viruses with a linear double stranded DNA molecule. Viruses belonging to the genus Mastadenovirus infect mammals, including six human adenovirus species (A to F) with more than 50 strains. Adenoviruses are prevalent in the human population; subclinical infections are common, and disease manifestations occur with infections of the respiratory, urinary, and digestive tracts as well as the eye.

***Asfarviridae*** are enveloped viruses with a double-stranded DNA genome. The family is comprised of a single species, which is the only known DNA virus dependent on transmission by an arthropod vector. The chief vector, the soft-bodied tick (Ornithodoros), transmits the **African swine fever virus (ASFV)** to its main hosts—wild and domesticated pigs—in which it causes a haemorrhagic fever with high mortality.

***Filoviridae*. Ebolavirus** is a relatively large virus with a size of 1–2 μm in length. It is enveloped and has a (-) sense single-stranded RNA genome. In humans it causes haemorrhagic fevers with severe and often fatal outcomes.

***Flaviviridae.* Hepatitis C virus (HCV)** is grouped within the *Flaviviridae,* which contain a positive-sense single-stranded RNA genome of approximately 9.6 kb. Chronic HCV infections are associated with an increased risk of liver-related morbidity and mortality.

***Hepadnaviridae*.** Hepatitis B viruses are small enveloped DNA viruses, which replicate via reverse transcription. Mammalian hepadnaviruses encode a polymerase, capsid proteins, envelope proteins, and the X-protein. Approximately 240 million people worldwide suffer from chronic infection with the **human hepatitis B virus (HBV)** and an estimated 780,000 die each year due to the acute or chronic consequences of hepatitis B, including hepatocellular carcinoma (HCC).

***Herpesviridae*** have a large (>100 kb) double stranded linear DNA genome within an icosahedral capsid surrounded by a protein layer (tegument) and an envelope. The *Alphaherpesvirinae* **herpes simplex virus (HSV) 1, HSV 2** and **varicella-zoster virus (VZV)** are major human pathogens, which can cause serious infectious illnesses ranging from cold sores and genital lesions to chickenpox, shingles, and encephalitis. **Kaposi’s sarcoma-associated herpes virus/human herpes virus 8 (HHV-8)** is a gammaherpesvirus with a strong association with different forms of Kaposi’s sarcoma. **Porcine pseudorabies virus (PRV)** causes a respiratory illness with a high mortality rate in piglets under one month of age.

***Iridoviridae.*** The viruses have double stranded DNA genomes, and infect predominantly invertebrates, but also some vertebrates (fish, amphibians, reptiles). Singapore Grouper Iridovirus (SGIV) is a member of the *Iridoviridae*.

***Orthomyxoviridae*** is a family of RNA viruses. The **influenza A virus** causes acute respiratory diseases in humans. Its genome is composed of eight negative-sense single-stranded RNA molecules encoding up to 11 proteins.

***Papillomaviridae*** is a large family of small, icosahedral, non-enveloped double-strand DNA viruses. Papilloma viruses are highly species specific. Several hundred types of papilloma virus are known that cause small, mostly benign, skin lesions (warts) in a wide range of mammals, snakes, turtles, and birds. Some types of papilloma virus also cause malignant tumours. As defined by the World Health Organization (WHO), there are 12 high-risk HPV types that cause cervical cancer [99]. Of particular interest here are **Human Papillomavirus (HPV) 16** and **HPV 18** which are linked to 70% of all human cervical cancers [100].

***Picornaviridae*** are non-enveloped viruses containing a single-stranded RNA genome with a positive sense orientation encoding a single long open reading frame. A member of this family, the **Foot-and-Mouth Disease virus (FMDV)** affects cloven-hooved animals, causing fever, blisters and—in some cases—death. Human infections with FMDV are a very rare occurrence. **Echovirus 1** is highly infectious, causing acute fevers particularly in children, as aseptic meningitis.

***Poxviridae*** are large enveloped viruses with linear, double-stranded, covalently-closed DNA molecules. The virions are pleomorphic with a lipoprotein surface membrane. Two versions of infectious particles are produced, mature virions (mVs) and extracellular virions (eVs), the latter possessing two additional membranes. **Vaccinia virus** can cause a mild febrile illness in adults, but is typically asymptomatic. Vaccinia virus has its most important use as the vaccination strain against smallpox. **Myxomavirus** causes a fatal disease in rabbits and was used to combat the feral rabbit populations in Australia. Smallpox, the first and to date only human disease to have been eradicated, was caused by a poxvirus.

***Retroviridae*. HIV** belongs to the **Lentiviruses** within the family ***Retroviridae***. UNAIDS and the WHO estimated that HIV, the causative agent for AIDS (Acquired Immune Deficiency Syndrome), killed more than 25 million people between 1981 and 2005, making it one of the most destructive pandemics in recorded history. HIV infects cells that play crucial roles in the human immune system, such as helper T cells (specifically CD4 + T cells), macrophages, and dendritic cells. The decline of CD4 + T cells severely compromises the immune system, exposing the patient to a wide variety of opportunistic microbial infections. Thus, the major causes of AIDS patients’ death are infections by a variety of bacteria, fungi, viruses, and associated malignancies. **Amphotropic murine leukemia virus (A-MLV),** a member of the genus gamma retrovirus, causes cancer in its murine host. The positive sense, single-stranded RNA virus acts as a model to study viral clearance and cancer development and is a possible vehicle in gene therapy.

***2.* *Bacteria***

***Bartonella bacilliformis*** is a Gram-negative bacterium that causes Carrions’ disease (bartonellosis). Upon transmission to humans via insect vectors (e.g., sand flies), it colonises human erythrocytes and subsequently invades vascular endothelial cells, leading to the formation of hemangiomatous dermal eruptions. One of the major virulence factors of *Bartonella* is the VirB/VirD4 type IV secretion system (T4SS), a multi-subunit transporter essential for establishing intraerythrocytic infection.

***Escherichia coli* K1** is the causative factor of meningitis in newborns.

**Enteropathogenic *E. coli* (EPEC)** is a major causative factor of diarrhoea in infants. The EPEC type III secretion system (T3SS) is one of the key virulence factors involved in the disruption of host intestinal homeostasis. This macromolecular transport machinery secretes multiple effector proteins into host intestinal epithelial cells, resulting in disruption of tight junctions and formation of the so-called attaching and effacing lesions.

***Helicobacter pylori*** is a Gram-negative motile microaerophilic bacterium that persistently colonises the stomach of approximately half of the population worldwide. It is the causative agent of chronic gastritis, peptic ulcer, and gastric cancer. *H. pylori* infection is believed to be the strongest identified risk factor of gastric cancer. *H. pylori* strains expressing a type IV secretion system (T4SS) are particularly virulent and are associated with strong pro-inflammatory responses and increased gastric cancer risk. The T4SS delivers the oncogenic protein CagA into the host cell. Translocated CagA exerts diverse effects on host cell functions such as cytoskeletal rearrangement and cellular elongation of the host cell, perturbation of cell-cell junctions, increased cell proliferation and altered pro-inflammatory responses.

***Neisseria gonorrhoeae*** is a Gram-negative bacterium that causes the sexually transmitted disease gonorrhoea.

**Non-typeable *Haemophilus influenzae* (NTHi),** a common commensal bacterium of the human nasopharynx, is capable of causing opportunistic mucosal infections. NTHi infection can result in diseases including otitis media, exacerbations of chronic obstructive pulmonary disease, conjunctivitis, and sinusitis. Of particular concern is a steady increase in NTHi-associated invasive diseases, including sepsis and pneumonia.

***Pseudomonas aeruginosa*** is a Gram-negative opportunistic human pathogen causing generalized inflammation and sepsis. It is a nutritionally highly diverse bacterium and can infect tissues that have reduced immunity or have sustained previous damages. Opportunistic colonisations of the critical organs can result in a fatal outcome. The bacterium can also cause cross-infections in hospitals and clinics, as it grows on moist surfaces as found on medical equipment.

***Salmonella* Typhimurium** is a Gram-negative bacterium of the intestinal lumen that causes gastroenteritis in humans and other mammals. Inflammation is a direct cause of host cell ruffling, which occurs as bacteria enter the epithelial cells lining the intestine. Symptoms caused by *S.* Typhimurium infection in mice resemble human typhoid fever. *Salmonella* Typhimurium encodes two T3SSs in discrete pathogenicity islands. One T3SS is encoded in the Salmonella pathogenicity island 1 (SPI-1). It modulates actin cytoskeleton dynamics and is required to mediate bacterial uptake into nonphagocytic cells. The other T3SS is encoded within SPI-2. Its expression is induced upon host cell entry. The SPI-2 T3SS is essential for bacterial replication within host cells.

***3.* *Parasitic Protists***

*Leishmania* parasites are kinetoplastids that cause a large spectrum of diseases, ranging from self-healing skin lesions to deadly visceral disease. Leishmaniasis, which is a vector-borne disease, affects 12 million people in more than 90 countries in tropical, subtropical and Mediterranean regions.

***Trypanosoma cruzi***, another kinetoplastid, is the etiological agent of Chagas disease, which affects millions of people in Latin America and poses a serious emerging threat to the US and other countries [101]. ***Plasmodium falciparum.*** Malaria affects millions of people worldwide and is still responsible for nearly 500,000 deaths each year, especially in children under five in sub-Saharan Africa. *Plasmodium falciparum* is the apicomplexan parasite responsible for the most severe form of the disease. Parasite transmission occurs through the bite of an infected mosquito. After an initial asymptomatic hepatocytic stage, parasites invade red blood cells undergoing cycles of asexual multiplication, which are responsible for the clinical symptoms of the disease. ***Plasmodium berghei*** causes malaria in rodents. It is a very valuable laboratory model for human malaria, since it enables investigation of the full life cycle, including the liver stage.

***Theileria*** parasites are veterinary pathogens transmitted by ticks, which cause debilitating and often-fatal diseases in cattle. The high economic impact of East Coast Fever, caused by *T. parva*, and—to a slightly lesser extent—tropical theileriosis, caused by *T. annulata*, warrants research efforts into the biology of these important pathogens. *Theileria* invade host leukocytes, inducing uncontrolled proliferation, apoptotic resistance and increased migration. Unlike most other apicomplexan parasites, *Theileria* cells are not surrounded by a parasitophorous vacuole but reside instead directly in the host cell cytoplasm. They can thus directly interact with host cell signalling molecules, making them a fascinating example of a eukaryotic pathogen able to significantly manipulate and transform its host cells [102].

***Toxoplasma gondii*** is the intracellular parasite responsible for toxoplasmosis, a disease affecting the human nervous system. Toxoplasmosis causes abortion and malformation in infected unborn children, neurological impairment in immunocompromised individuals, and has been associated with behaviour alterations, and schizophrenia. The parasites develop in a parasitophorous vacuole (PV) within a variety of host cells. ***Cryptosporidium parvum****,* another pathogenic apicomplexan parasite, causes gastrointestinal disease in humans after ingestion as a cyst and subsequent invasion of intestinal epithelial cells. Disease is usually self-limiting in otherwise healthy individuals but can cause severe problems in immunocompromised patients.

#### 2.1.2. Pathogen Entry into the Host by Pathways Independent of Macropinocytosis

A number of pathogens have evolved to exploit host pathways distinct from classical macropinocytosis to enter host cells, yet these pathways often also involve PAK1 activity. Host cell entry by human papilloma virus type 16 (HPV16) occurs via clathrin-, caveolin-, and lipid-raft independent endocytosis. HPV16 uses a possible novel entry pathway, which shares characteristics of macropinocytosis by depending on actin dynamics, tyrosine kinase signalling, protein kinase C (PKC), and PAK1 activity. The entry process is distinguished from classical macropinocytosis by differences in cholesterol sensitivity, GTPase requirements and the formation of small uncoated vesicles [103,104]. Human immunodeficiency retrovirus type 1 (HIV-1) follows a non-classical macropinocytosis pathway of entry into macrophages, which depends on intact lipid rafts, Na^+^/H^+^ exchange, dynamin, Rac 1 and PAK1, but not on PI3K and myosin II [105], and requires CCR5 engagement [106]. Cofilin is phosphorylated and inactivated to facilitate early entry of HIV, a process that requires actin polymerization. Cofilin is phosphorylated by LIM kinase 1, which is activated by the HIV type 1 (HIV-1) gp120 envelope protein mediated through the Rac1 and PAK pathway [34].

The activity of PAK1 also contributes to the internalisation of the bacterium *Pseudomonas aeruginosa* into host cells, as supported by two lines of evidence [107]: first, siRNA downregulation of PAK1 expression prevents the entry of *P. aeruginosa* strain K into HeLa cells [107]; second, ectopic expression of a kinase-dead or a constitutively active mutant of PAK1 significantly impairs *P. aeruginosa* internalisation into MDCK cells [107], suggesting that oscillatory activation and inactivation of PAK1 are important for *P. aeruginosa* internalisation. The molecular mechanism by which *P. aeruginosa* activates PAK1 remains unclear. In contrast to many other pathogenic bacteria that activate PAK1, *Escherichia coli* K1 inactivates PAK1 [52]. Upon infection of human brain microvascular endothelial cells (HBMEC), cells that form the blood-brain barrier, *E. coli* K1 induces a reduction in PAK1 activity, resulting in upregulation of myosin light chain kinase (MLCK) activity and increased light chain of myosin II (MLC) phosphorylation. Phosphorylated MLC recruited to the site of bacterial entry may then facilitate actin-mediated internalisation. These findings emphasize that even though there is a surprising conformity in the role of PAK1 in bacterial pathogenesis, it is by no means ubiquitous. Thus one must exercise caution when attempting to extrapolate what is known about the role of PAK1 from one type of pathogenic infection to another. Internalization of non-typeable *Haemophilus influenzae* (NTHi) strain 375 into human lung epithelial cells requires microtubule polymerization and the activity of PI3K [53]. PAK1 mediates Rac1-driven microtubule polymerization [108], while functioning as a negative regulator of the microtubule-destabilizing protein Op18/stathmin [55]. NTHi infection results in activation of Rac1 and PAK1, which then leads to inhibition of the microtubule-destabilizing protein Op18/stathmin, favouring microtubule polymerization [54].

Actin polymerisation within the host cell is also essential for phagocytosis of the amastigote form of the protist *Leishmania*, as demonstrated in a *Drosophila* model [109]. *L. donovani* amastigote uptake by phagocytic cells is Rac1-dependent, while the uptake of *L. amazonensis* into Chinese hamster ovary (CHO) cells as well as *L. donovani* into Drosophila S2 cells was shown to be Cdc42-dependent [109,110,111]. PAK1 is, as mentioned previously, one of the main effectors of Rac1 and Cdc42-driven actin polymerisation. The apicomplexan parasite *Toxoplasma gondii* critically depends on host cell signalling to alter essential processes in its host cell, such as transcription, trafficking, cytoskeleton dynamics, and metabolism [112,113,114,115]. Particularly relevant in this context, successful invasion by *T. gondii* mobilises the actin network of host cells [116,117]. As noted above for kinetoplastids (which include *Leishmania* and *Trypanosoma*), active Rac1 was shown to accumulate on the newly formed parasitophorous vacuole membrane following invasion by *T. gondii* of COS-7 cells (monkey kidney cells) [118]. The authors of this study point to PAK1 being the likely downstream kinase of Rac1 [118]. The invasion of host erythrocytes by merozoites of the apicomplexan parasite *Plasmodium falciparum,* in contrast, does not seem to depend on the actin activity of the host, as pre-treatment of erythrocytes with the actin polymerisation inhibitor Cytochalasin D did not affect invasion [119]. Chemical inhibition of the PAK-effector MEK1 likewise had no effect on the invasion of erythrocytes by *P. falciparum* merozoites, but blocked subsequent parasite development [59]. *P. berghei* sporozoites, however, were shown to require formation of host cell F-actin for the invasion of hepatocytes. The abolishment of actin dynamics by two different actin-destabilizing drugs—jasplakinolide and mycalolide B—significantly impaired host cell invasion by *P. berghei* sporozoites as well as *T. gondii* tachyzoites [116]. It remains to be shown if the involvement of actin dynamics during *P. berghei* sporozoite invasion is dependent on PAK1 function. Invasion of epithelial cells by the apicomplexan parasite *Cryptosporidium parvum* (Iowa strain) requires the recruitment of the host actin cytoskeleton to the apical membrane of epithelial cells. Remodelling of the host cytoskeleton is initiated by PI3K-activated Cdc42 [120]. To date, an involvement of PAK1 as a possible effector of Cdc42 in *C. parvum*-infected cells has not been documented, but is in our view highly likely.

### 2.2. PAK1-Dependent Manipulation of Host Cell Cytoskeleton by Pathogens

Successful infection of a host cell requires entry and post-entry events that exploit normal cellular functions to optimize replication and production of the invading pathogen. The dynamics of actin microfilaments determines cellular shape and movement, phagocytosis, intercellular communication, and organelle dissemination, and therefore represent a prime target for many invading pathogens. The following section delineates how pathogens manipulate the host cell cytoskeleton in cellular processes following invasion.

Alphaherpesvirinae encode US3 kinase, a serine/threonine kinase with sequence similarities to group I PAKs, indicating a possible phylogenetic relationship [121]. The sequence similarity between US3 kinase and group I PAKs is only approximately 25%, but both the US3 kinase and group I PAKs possess a N-terminal regulatory domain, a C-terminal catalytic domain, and a cluster of approximately 100 acidic amino acids, which is located upstream of a conserved subdomain [121]. The US3 kinase is a multifunctional protein that has important roles in virus virulence, and is involved in inhibition of apoptosis, virion morphogenesis, and interference with host immune responses [40,41]. In porcine pseudorabies virus (PRV), US3 kinase modulates the Rac1/Cdc42 signalling pathway in a swine testicular cell line and mouse embryo fibroblasts by activating the downstream effectors, namely, group I PAKs, resulting in the induction of dramatic rearrangements in the organization of the actin cytoskeleton, leading to the breakdown of actin stress fibres and the formation of actin dependent cell projections [19]. US3 kinase mediates the actin re-organization by binding and phosphorylating group I PAKs; PAK1 is required for US3-mediated formation of cell projections (see BOX 2 for PAK2 involvement) [19]. The use of IPA-3, an inhibitor of group I PAKs that prevents US3-mediated activation of group I PAKs, interfered with the US3-mediated activation of cofilin, an important actin-binding factor essential for the reorganization of actin filaments [43]. The cytoskeletal changes are associated with enhanced intercellular virus spread [18]. Preventing the US3-mediated activation of group I PAK by the inhibitor IPA-3 reduced the PRV spread in the epithelium of porcine respiratory mucosa explants, reduced the invasion across the basement membrane, which represents the barrier between the epithelium and the lamina propria. The presence of the PAK inhibitor IPA-3 reduced the plaque number that breached the basement membrane [122]. In endothelial cells infected with Human herpes virus 8 (HHV-8), Rac1 and PAK1 activity was increased. PAK1 mediated disassembly of cell junctions and enhanced vascular permeability by phosphorylating vascular endothelial (VE)-cadherin and β-catenin, proteins that are involved in maintaining the integrity of cell junctions [39]. In contrast, Marek’s disease virus (MDV), also a member of the Alphaherpesvirinae, did not induce cellular extensions in primary chicken embryo skin cells (CESC), and Rac-PAK signalling had an inhibitory effect on MDV cell-to-cell spread in CESCs [123]. In addition to the PAK1 requirement for the Vaccinia virus (family *Poxviridae*) entry into the host cell, PAK1 also seems to play an important role for virus dissemination. Knock out PAK1 (PAK1^−/−^) mouse embryonic fibroblasts allowed the formation of cell-associated enveloped virions (CEV), but the formation of actin tails was compromised, resulting in reduced viral plaque sizes and reduced viral dissemination. Upon vaccinia virus infection, ARPC1, the regulatory component of the actin polymerization complex ARP2/3, is not phosphorylated in the absence of PAK1 compromising the formation of actin tails and viral dissemination. Consistent results were obtained with monkey kidney epithelial cells (BSC-40) treated with the PAK1 inhibitor IPA-3 [124].

**Figure 3:**
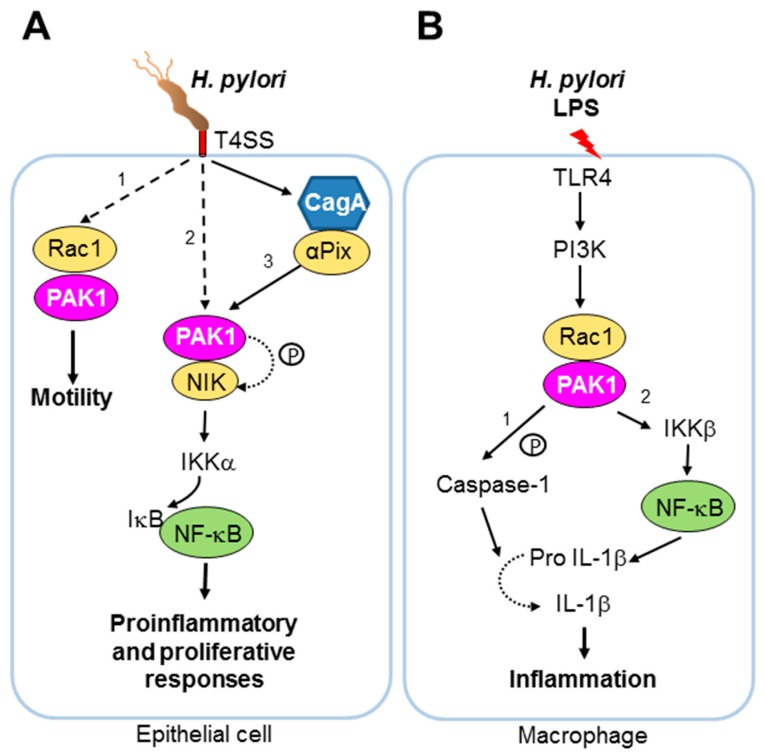
The bacterium *Helicobacter pylori* is a well-studied example of a pathogen manipulating host PAK1 signalling. (**A**) Upon infection of human epithelial cells, *H. pylori* activates Rac1 and stimulates recruitment of PAK1 to active Rac1 in a T4SS-dependent manner (pathway 1). *H. pylori* also induces interaction between PAK1 and NIK (pathway 2). Such interaction and subsequent phosphorylation of NIK by PAK1 have been proposed to be required for NF-κB activation in *H. pylori* infection [48]. Moreover, *H. pylori* is capable of stimulating NF-κB activation in a PAK1-dependent pathway through αPix (pathway 3). NF-κB activation and recruitment of PAK1 to active Rac1 both appear to require the T4SS, a complex macromolecular transporter and key virulence factor of *H. pylori* [48] (see BOX 2). CagA, the protein cargo of T4SS, has been shown to form a complex with αPix during *H. pylori* infection [137]. The T4SS is shown in red. Phosphorylation is indicated by the symbol “p”. Proteins identified to interact with PAK1 in *H. pylori*-infected epithelial cells are shown in yellow. (**B**) Upon stimulation of the human macrophage cell line, THP-1, purified *H. pylori* LPS can induce IL-1β secretion in a TLR4-dependent manner via post-translational (1) and transcriptional (2) pathways [138]. In the former pathway, *H. pylori* LPS stimulates Rac1 activity and the kinase activity of PAK1, triggering interaction of PAK1 with caspase-1 and possibly the subsequent phosphorylation of caspase-1 at S376 by PAK1. Phosphorylated caspase-1 then cleaves pro-IL-1β into mature active IL-1β. *H. pylori* LPS can also activate IL-1β expression at the transcriptional level via NF-κB-activation in a manner that requires the kinase activities of both PAK1 and IKKβ. It has been proposed that the up-regulation of IL-1β expression by *H. pylori* LPS involves the PI3K/Rac1/PAK1 signalling axis [138]. In-depth understanding of the molecular mechanism by which the kinase activity of PAK1 is modulated in these various pathways is warranted.

PAK1 also orchestrates the enhanced actin-dependent migratory responses in the host cell that are triggered upon infection by bacterial pathogens. During infection of the human gastric epithelial cell line AGS, *H. pylori* (strains P1 and P12) stimulates Rac1 activity and the recruitment of Rac1 to the host membrane at bacterial attachment sites, concomitant with increased motility of the host cell [51]. Transient recruitment of PAK1 to active Rac1 occurs as soon as one hour after infection with wild-type *H. pylori*, whereas recruitment of PAK1 or Rac1 activation was not observed with a *H. pylori* mutant lacking the type IV secretion system (T4SS) [51] (Figure 3). How the *H. pylori* T4SS regulates PAK1-Rac1 interaction and downstream signalling remains to be further investigated. The Enteropathogenic *E. coli* (EPEC) type III secretion system (T3SS), a macromolecular transport machinery, is one of the key virulence factors involved in the disruption of host intestinal homeostasis [125] (see BOX 3). One of the EPEC type III secretion effector proteins, EspG, interacts with the Rac1/Cdc42 binding domain of PAK1 [31]. While the effect of this interaction on the kinase activity or substrate-binding affinity of PAK1 remains unclear, binding of EspG to PAK2 has been shown to result in a 7.6 ± 2.5-fold increase in the kinase activity of PAK2 [32]. Data from crystallographic studies suggest that EspG activates PAK2 by perturbing the interface between the auto-inhibitory domain and the kinase domain of PAK2 [32]. Given the pivotal role of PAK kinases in actin remodelling and signal transduction downstream of small GTPases, the interaction of EspG with PAK1 and/or PAK2 (see BOX 2 for PAK2) is likely to have important implications for EPEC pathogenesis. It is tempting to speculate that the interaction with PAKs may enable EspG to fine-tune host cytoskeletal dynamics and/or vesicle trafficking to favour bacterial invasion and tissue penetration during infection. Likewise, during *Bartonella bacilliformis* infection of human endothelial cells, Rac1, Cdc42 and PAK1 are activated, accompanied by the formation of filopodia and lamellipodia in the host cells [45].

To our knowledge, there has been no investigation into the role of host cell PAK during *Leishmania* infection. However, it has been established that accumulation of F-actin plays a role in preventing the maturation of intracellular phagolysosomes in murine macrophages, thereby sustaining infection [126]. This accumulation in murine macrophages was found to be dependent on the activity of the small Rho GTPases Cdc42 and Rac1 [127], suggesting the involvement of a PAK1-dependent pathway. Actin cytoskeleton re-organization triggered by parasites is not always PAK1-dependent; for example, even though host actin filament integrity is essential for *T. gondii* (RH strain) to egress from the parasitophorous vacuole, PAK1 only seems to play a minor role in parasite egress, as suggested by the observation that parasite egress is diminished by only 15% following treatment with the PAK1 inhibitor IPA-3 [128]. While investigating matrigel invasion by bovine macrophage-derived lines infected with the apicomplexan *Theileria annulata*, Ma et al. found that Cdc42 was more activated in aggressively invasive *Theileria* TaH12810 cells than in culture-attenuated *Theileria* Thei cells [129], suggesting a role for Cdc42 activation in motility and thus disease virulence. The authors make no comment on a possible role of PAK1 as the effector of Cdc42-activation. However, considering activated Cdc42 was pulled-down using PAK1-agarose [129], it is clear that Cdc42 is in a state that can bind PAK1. It is thus likely that PAK1 plays a role in the virulence of *T. annulata* infections.

### 2.3. PAK1-Dependent Manipulation of Host Cell Apoptosis by Pathogens

The invasion of cells by pathogens triggers cellular defence mechanisms that can lead to apoptosis; this has placed strong evolutionary pressure on pathogens to develop systems that interfere with apoptosis of their host cell to ensure their own survival [130,131,132]. Multiple apoptotic pathways in human cells are associated with PAK1 activity, particularly through the phosphorylation of BAD (Bcl-2 antagonist of cell death), which inhibits apoptosis by releasing the anti-apoptotic molecule Bcl-2. BAD can be phosphorylated in a pro-survival configuration either directly by PAK1 or by PAK1-activated Raf-1 [133]. To our knowledge, nothing is known about bacteria influencing PAK signalling to prevent host cell death. However, accumulating data suggest that several viruses and parasites have developed ways to hijack PAK1 in triggering pro-survival and anti-apoptotic events; some of the key examples will be discussed below.

The multifunctional US3 serine/threonine kinase encoded by the *Alphaherpesvirinae* [40,41] (see previous paragraph) exerts anti-apoptotic activity, as exemplified by the pseudorabies virus. US3 kinase has the ability to directly phosphorylate and inactivate pro-apoptotic proteins (Bad, Bid), but also interferes with apoptosis through PAK1, generating partly redundant pathways [42]. In addition to its ability to prevent apoptotic cell death, pseudorabies US3 kinase protects infected cells from lysis mediated by natural killer (NK) cells. US3-mediated enhancement of binding to the inhibitory CD300a NK receptor depends on the modulation of the phosphatidylserine on the cell surface and on group I PAK signalling pathway [134]. Among the *Retroviridae*, the multifunctional HIV-1 Nef protein (negative factor) has an anti-apoptotic effect. Phosphatidylinositol 3-kinase (PI3-kinase) is required for Nef-dependent PAK activation and phosphorylation, and ensuing inactivation of the pro-apoptotic Bad protein. Nef-induced Bad phosphorylation can be mediated by PAK1 [30]. The X-protein (HBx) of Hepatitis B Virus (family *Hepadnaviridae*) is a small multifunctional protein, with the ability to modulate several signalling pathways that directly impact the development of hepatocellular carcinoma (HCC) [35]. HBV infection results in an enhancement of PAK1 transcription, which confers resistance of hepatoma cells to anoikis, a form of apoptosis that involves cells detaching from their extracellular matrix [37]. Dysregulation of PAKs play an important role in HCC progression and metastasis, and the HBx protein seems to be the driver of elevated PAK1 expression levels, which are positively correlated with poor prognosis [36,37].

NF-κB is a protein complex that acts as a transcription factor and is involved in the regulation of a large number of genes with importance for immune and inflammatory responses, cell growth, apoptosis, and transformation. The inhibitory IκB protein sequesters the NF-κB complex in the cytoplasm. In response to various stimuli, IκB is phosphorylated and inactivated by the IκB kinases IKKα and IKKβ, which allows the release of NF-κB, its translocation to the nucleus and its binding to regulatory DNA sequences. Of particular relevance here is that Rac1 and Cdc42 and their PAK effector molecules can also activate NF-κB by an IKK-independent mechanism in murine macrophages and fibroplasts [62].

While *Toxoplasma gondii* is known to inhibit host cell apoptosis via NF-κB, there are no available data on the mechanism of NF-κB activation in this case [135,136]. Given that PAK1 is a known NF-κB activator in other systems (see next paragraph), it is however plausible that PAK1 might be involved in NF-κB activation by *T. gondii*. The kinetoplastid parasite *Trypanosoma cruzi* survives in large numbers in the cytosol of a number of different cell types. The trans-sialidase PDNF (parasite-derived neurotrophic factor), a parasite surface protein shed into host cytoplasm, triggers an anti-apoptotic response in the host cell by activating Akt, inhibiting gene-expression of proapoptotic proteins and increasing the expression of Akt in the *T. cruzi* Silvio strain [57]. Nothing is known about the involvement of PAK1 in this long-term survival mechanism of the parasites. However as PAK1 is known to be involved in Akt activation, by acting as a kinase-independent scaffold in the PI3K-AKT-mTOR pathway, it is conceivable that PAK may also play a role in the anti-apoptotic strategy of *T. cruzi* [58].

### 2.4. PAK1-Dependent Manipulation of Host Cell Early Nuclear Response by Pathogens

Human Herpesvirus 8 (HHV-8) encodes a G protein-coupled receptor (GPCR), an oncogene that has been implicated in the pathogenesis of Kaposi’s sarcoma. Signalling from the HHV-8 encoded GPCR is ligand-independent, and results in the activation of PAK1 through Rac1 and Cdc42. PAK1 activation by the virus-encoded GPCR results in an increase of activated IKKβ which phosphorylates IκB and thus activates NF-κB. This represents a possibly important step for HHV-8 mediated cellular transformation [38].

Activation of NF-κB plays a pivotal role in *Helicobacter pylori*-induced inflammatory responses [47], in which PAK1 has emerged to be an important player. Pronounced activation of PAK1 by *H. pylori* (strains P1 and P12) upon infection of the gastric adenocarcinoma cell line AGS occurs 45 min after infection [48,49]. As mentioned above, activation of PAK1 is mediated by the *H. pylori* T4SS through activation of Rac1 [51] (see BOX 3). The role of PAK1 in NF-κB activation by *H. pylori* was highlighted by the observations that expression of a kinase-inactive construct of PAK1 in *H. pylori*-infected cells blocks NF-κB transcriptional activity, and that interaction between PAK1 and the NF-κB -inducing kinase (NIK) is stimulated in *H. pylori*-infected cells, accompanied by phosphorylation of NIK by PAK1 [48]. NIK plays an important role in the NF-κB activation pathway by phosphorylating and activating IKKs [50], which are downstream effectors of PAK1 [48]. Furthermore, both kinase-inactive NIK and kinase-inactive PAK1 block *H. pylori*-induced IKK phosphorylation and NF-κB transcriptional activity [48,49], providing further evidence that PAK1 plays a crucial role in NF-κB activation by *H. pylori* (Figure 3).

*Bartonella bacilliformis*-stimulated PAK1 activation occurs by 1 h after infection of human endothelial cells. PAK1 subsequently becomes inactivated at 2 h and 4 h post-infection, and is then activated again by 12 h post-infection. Interestingly, this parallels the temporal pattern of the activation of JNK and activation of the transcription factor AP-1 during *B. bacilliformis* infection [45]. As discussed in the paragraph on macropinocytosis, involvement of PAK1 in the activation of JNK has been observed during *S. typhimurium* strain SL1344 invasion of COS-1 cells [86]. It is thus tempting to speculate that the JNK activation observed during *B. bacilliformis* infection might also be driven by PAK1 activation. The use of appropriate inhibitors, siRNAs or negative-dominant constructs of PAK1 will be instrumental in testing this hypothesis. *Neisseria gonorrhoeae* infection of host epithelial cells stimulates activation of AP-1, another transcription factor involved in nuclear response pathways. AP-1 activation by *N. gonorrhoeae* results in the induction of a series of inflammatory responses [46] and is dependent on the activities of small Rho GTPases and JNK [33]. Given that PAK1 is a downstream effector of the small Rho-GTPases Rac1 and Cdc42, the role of PAK1 in such a signalling cascade has been investigated. Naumann et al. showed that ectopic expression of the dominant-negative kinase-dead mutant of PAK1 in HeLa cells significantly inhibits AP-1 activation in *N. gonorrhoea*-infected cells [33], suggesting that PAK activity is required for AP-1 activation in this system. These findings further highlight the recurrent theme that PAK1 plays a pivotal role in not only actin cytoskeletal rearrangement but also the activation of host nuclear responses in certain bacterial infections.

It is well established that NF-κB is constitutively activated in *Theileria*-infected leukocytes. The parasite recruits the IκB kinase (IKK) signalosome complex to the macroschizont surface by an unknown mechanism and activates IKK. Active IKK phosphorylates IκB, marking it for degradation and thus sets NF-κB free to translocate into the host cell nucleus and regulate target genes. Durrani et al. showed that PAK1 expression was up-regulated 22-fold in *T. annulata* infected cells, when compared to uninfected bovine leukocytes [61]. In view of these findings and of the fact that activation of NF-κB in (murine) fibroblasts and macrophages can be dependent on PAK1 activity [62], we propose that investigating the role of host PAK1 during *Theileria*-infection is likely to yield interesting results and novel insights into the molecular pathogenesis of *Theileria* infection.

### 2.5. Host Cell PAK1 Activity Plays a Crucial Role in Pathogen Replication and Development

Some pathogens live and replicate within their respective host cells, while others—in particular many bacterial pathogens discussed in this review—associate closely with host tissues but do no invade host cells. For several members of both groups the modification of human signalling pathways involving PAK1 plays an important role during pathogen development.

In the case of Influenza A virus (family *Orthomyxoviridae*), constitutively active PAK1 induced higher viral titres, whereas siRNA knockdown of PAK1 resulted in a reduction of titres [44]. Human lung epithelial A549 cells infected with Influenza A virus showed an induction of ERK activity as well as PAK1 stimulation. This suggests the possibility of a functional relationship between PAK1 activation and the MAPK/ERK pathway, through PAK1 activation of the ERK-activator MEK1 [44], similar to observations in *Plasmodium*-infected erythrocytes, as detailed below [59]. Replication of Hepatitis C virus (HCV) (family *Flaviviridae*) is impaired in the presence of activated PAK1. PAK1-specific knock down in HCV-infected cells enhanced viral RNA production. The serine/threonine kinase mTOR controls PAK1 activation, which mediates the suppression of HCV replication. Ishida et al. suggest that PAK1 could influence HCV replication through the regulation of cytoskeleton components [10].

Recently a role of PAK1 was implicated in *Salmonella* replication in the host cell [56]. SopB, SopE and SopE2, effector proteins of the SPI-1 type III secretion system (T3SS) of *S. typhimurium*, activate small GTPases, which in turn activate PAK. Results of inhibitor studies suggest that PAK then activates the Src-family tyrosine kinase, c-Abl, resulting in activation of the cytoplasmic transcription factor, signal transducer and activator of transcription 3 (STAT3) and subsequent modulation of host gene expression profile. This signalling axis is important for the formation of the specialized bacteria-containing membrane-bound compartments known as *Salmonella*-induced filaments (SIFs) and the intracellular growth of *S. typhimurium* in these compartments [56].

*Plasmodium falciparum* multiplies in mature erythrocytes, a process requiring host cell G protein-coupled receptor signalling for both invasion and development [139,140]. PAK1 was shown to activate human MEK1 in erythrocytes upon infection with *Plasmodium falciparum* (3D7) and *P. berghei* [59]. The PAK inhibitor IPA-3 blocked parasite development at the trophozoite stage, demonstrating the essential role of human PAK1 in intra-erythrocytic *Plasmodium* development [59]. Inhibition of the PAK1-effector MEK1 similarly affected the development of the rodent malaria parasite *P. berghei* in both murine erythrocytes and human hepatocytes [59,60]. Consistent with this notion, a kinome-wide siRNA knock-down screen showed that down-regulation of PAK3 and the scaffold protein MAPKSP1, which enhances MEK activation, impaired infection of human hepatocytes by *P. berghei* [59,141]. Curiously, no phosphorylation of the MEK1 substrates ERK1/2 could be detected in *Plasmodium*-infected erythrocytes, suggesting that the PAK1-MEK1 pathway acts on a different effector, which could be of either host or parasite origin [59]. Given that the parasites themselves possess no homologues of PAK1 and MEK1, as shown by phylogenetic analysis of sequences conforming to protein kinase HMMs (see for example [142]), we are currently investigating how erythrocytic PAK1 is activated upon *Plasmodium* infection, and what role the pathway plays for the development of the parasites.

## 3. Conclusions and Future Prospects

PAKs constitute a group of kinases of paramount importance in mammalian cell biology. The multiple central functions that PAK paralogues play in various process biological processes such as cytoskeleton dynamics, cell motility, proliferation, and apoptosis make them a target of choice for manipulation by intracellular pathogens, all of which rely on the host cell machinery to ensure their own entry, survival, proliferation, and exit. Our knowledge of how pathogens manipulate host cell signalling pathways to their own advantage has expanded significantly in recent years. In this review we have detailed the current understanding of the molecular mechanisms by which a range of different viral, bacterial and protozoan pathogens manipulate host PAK1 to a multitude of effects that are beneficial for their proliferation and survival. The signals transmitted by PAK1 regulate a wide variety of processes during all stages of an infection or a pathogen’s life cycle in its human host. How the signal cascades involving PAK1 are influenced by pathogens remains to be elucidated in detail in most instances. There is an astonishing unicity in the way pathogens from widely different taxons handle the control of many aspects of their host cell’s responses, yet the wide variety of responses require in-depth research for each taxon independently. Thorough investigation of human PAK1 activation in response to pathogens is likely to provide significant insights into the role of PAK1 as a potential therapeutic target. Inhibition of PAKs as an anti-cancer therapeutic strategy has attracted increasing interest since the late 1990s, with the pan-PAK inhibitor, PF-3758309, being the first PAK inhibitor to enter clinical trials on cancer patients [9]. Experiments in a mouse model of fragile X syndrome further showed that expression of an auto-inhibitory domain of PAK alleviates some symptoms in the affected mice, suggesting that PAKs could also be promising drug targets in diseases other than cancers [9]. As can be expected from the multiplicity of function of PAK paralogues, toxicity is an issue; for example, animal studies with the selective group I PAK (pan-PAK1, 2, 3) inhibitor G-5555 uncovered acute cardiovascular toxicity with a narrow therapeutic window [143]. Nevertheless, in view of the central role of PAK signalling pathways in the pathogenesis of diverse microbial infections, we propose that PAK inhibitors might represent valuable new leads in the treatment of a wide range of infectious diseases.

We hope that this review will generate incentive for anti-infective drug development using host PAKs as a target. From the pharmacological perspective, drug development targeting PAKs has been slow, due to a large ATP binding pocket with high conformational flexibility, which renders the design of ATP-competitive inhibitors very challenging. The development of allosteric inhibitors on the other hand, though still in its early stages, shows potential and is likely to provide promising lead compounds [9,144,145]. Outcomes of PAK allosteric inhibitor development are eagerly awaited, and may have tremendous impact on human and animal health not only in the context of drug discovery in cancer and inflammation, but also in the context of much needed anti-infectives with untapped modes of action.

## Figures and Tables

**Table 1:** Activation of signalling pathways utilizing PAK1 by pathogens; only pathways for which two or more components are known were included in this table.

Name of Pathogen	Pathogen Type	Effect on PAK1	Pathogen Mediator	Involved Pathway Components	Outcome	Pathogen Process	References
HIV	RNA virus	Activation	Nef	PAK1, PAK2, PI3K, BAD	Anti-apoptotic	Pathogen survival	[22,23,24,25,26,27,28,30]
		Activation	gp120	Rac1, PAK1, LIMK1	Actin polymerization	Invasion	[34]
HBV	DNA virus	Upregulation	HBx	PAK1	Anti-apoptotic (Anti-Anoikis)	Pathogen survival	[35,36,37]
Human herpes virus 8 (HHV-8)	DNA virus	Activation	GPCR	Rac1, Cdc42, PAK1, IKKβ, IκB, NF-κB	Cellular transformation to Kaposi’s sarcoma	Pathogen survival	[38]
			Not known	Rac1, PAK1, (VE)-cadherin, β-catenin	Disassembly of cell junctions, enhanced vascular permeability	Cytoskeletal changes independent of invasion	[39]
Alphaherpes-virus (e.g., Herpes simplex virus, Pseudorabies virus)	DNA virus	Activation	US3	PAK1, Bad, Bid	Anti-apoptotic, protection against NK cells	Pathogen survival	[40,41,42]
		Activation	US3	PAK1, cofilin	Breakdown of actin stress fibres, formation of actin dependent cell projections	Cytoskeletal changes independent of invasion	[19,43]
Influenza A	RNA virus	Activation	Not known	PAK1, MEK1	Higher viral titres	Pathogen development	[44]
EPEC	Bacteria	Activation	T3SS effector protein EspG	PAK1, PAK2	Not known	Not known	[31,32]
*B. bacilliformis*	Bacteria	Activation	Not known	Rac1, Cdc42, PAK1	Formation of filopodia and lamellipodia	Cytoskeletal changes	[45]
*N. gonorrhoeae*	Bacteria	Activation	Not known	AP-1, JNK, Rac1, Cdc42, PAK2, PAK1	Inflammatory response	Host nuclear response	[33,46]
*H. pylori*	Bacteria	Activation	T4SS effector protein CagA	PAK1, NIK, IKKs, NF-kB	Inflammatory responses	Host nuclear response	[47,48,49,50]
		Activation	T4SS effector(s)	Rac1, PAK1	Increased motility of host cell	Cytoskeletal changes independent of invasion	[51]
*E. coli* K1	Bacteria	Inactivation	Not known	MLCK, PAK1	Actin-mediated internalisation	Invasion	[52]
Non-typeable *H. influenza* (NTHi)	Bacteria	Activation	Not known	Rac1, PI3K, PAK1, Op18/stathmin	Microtubule polymerization	Invasion	[53,54,55]
*S. Typhimurium*	Bacteria	Activation	T3SS effectors proteins SopB, SopE, SopE2	GTPases, PAK1, c-Abl, STAT3	Intracellular growth of the pathogen	Pathogen development	[56]
*T. cruzi*	Parasite	Activation?	PDNF	PAK1, Akt	Anti-apoptotic	Pathogen survival	[57,58]
*P. falciparum,* *P. berghei*	Parasite	Activation	Not known	PAK1, MEK1	Parasite survival	Pathogen development	[59,60]
*T. annulata*	Parasite	upregulation	Not known	IKK, IκB, NFκB, PAK1	Host cellular transformation	Host nuclear response	[61,62]

**Table 2:** Pathogen entry into host cells by PAK1-mediated macropinocytosis; all signalling components listed are human proteins, unless otherwise noted.

Pathogen	Type of Pathogen	Cell Factor (Receptor)	Known Pathway Components	Effector of PAK1/Function	References
A-MLV	RNA virus		Rac1, PAK1, RhoG		[68]
Echovirus 1	RNA virus	α2β1 integrin	PI3K, PLC, PKCα, Rac1, PAK1	CtBP-1/BARS, Macropinosome closure	[64,69]
FMDV	RNA virus	αvβ1, αvβ3, αvβ6, αvβ8 integrin	RTK, Rac1, PAK1, myosin II, PKC		[70]
Ebola virus	RNA virus	C-type lectin, phosphotidylserine receptor	PAK1	bCtBP-1/Bars	[72,73]
Influenza A virus	RNA virus	Sialic acids	PAK1, Src		[74]
SGIV	DNA virus		PAK1, Rac1		[75]
ASFV	DNA virus	EGFR	PI3K-Akt, Rac1, PAK1		[76]
HAdV-3	DNA virus	CD46 and integrins	PAK1	CtBP-1/fission and stabilization of macropinosome	[77,78,79]
Vaccinia virus	DNA virus	MARCO, phosphotidylserine receptor	Cdc42 or Rac1, PAK1	Actin rearrangement, formation of filopodia, membrane blebbing	[67,80,81]
*S.* Typhimurium	Bacteria	Type III secretion system stimulates macropinocytosis; no host cell entry occurs	SopE (*S.* Typhimurium protein), JNK, PAK1	JNK activation, membrane ruffling	[85,86]
*T. cruzi*	Parasite		Rac1, PAK1		[95,96]
